# What is the optimal isodose line for stereotactic radiotherapy for single brain metastases using HyperArc?

**DOI:** 10.1002/acm2.14408

**Published:** 2024-06-11

**Authors:** Tomohiro Sagawa, Toshiki Ikawa, Shingo Ohira, Naoyuki Kanayama, Yoshihiro Ueda, Shoki Inui, Masayoshi Miyazaki, Koji Konishi

**Affiliations:** ^1^ Department of Radiation Oncology Osaka International Cancer Institute Osaka Japan

**Keywords:** brain metastases, HyperArc, isodose line, stereotactic radiotherapy, treatment planning

## Abstract

**Purpose:**

The study aimed to investigate the optimal isodose line (IDL) in linear accelerator‐based stereotactic radiotherapy for single brain metastasis, using HyperArc. We compared the dosimetric parameters for target and normal brain tissue among six plans with different IDLs.

**Methods:**

This study included 30 patients with single brain metastasis. We retrospectively generated six plans for each tumor with different IDLs (80%, 70%, 60%, 50%, 40%, and 33%) using HyperArc. All treatment plans were normalized to the prescription dose of 35 Gy in five fractions which was covered by 95% of the planning target volume (PTV), defined by adding a 1.0 mm margin to the gross tumor volume (GTV). The dosimetric parameters were compared among the six plans.

**Results:**

For GTV > 0.1 cm^3^, the ratio of brain–GTV volumes receiving 25 Gy to PTV (V_25Gy_/PTV) was significantly lower at IDL 40%–70% than at IDL 80% and 33% (*p* < 0.01, retrospectively). For GTV < 0.1 cm^3^, V_25Gy_/PTV decreased continuously as IDL decreased. The values of D_99%_ and D_80%_ for GTV increased with decreasing IDL. An IDL of 50% or less was required to achieve D_99%_ of greater than 43 Gy and D_80%_ of greater than 50 Gy. The mean values of D_99%_ and D_80%_ for IDL 50% were 44.3 and 51.9 Gy.

**Conclusion:**

The optimal IDL is 40%–50% for GTV > 0.1 cm^3^. These lower IDLs could increase D_99%_ and D_80%_ of GTV while lowering V_25Gy_ of normal brain tissue, which may help reduce the risk of radiation necrosis and improve local control.

## 1 INTRODUCTION

The main goal of stereotactic irradiation for brain metastases is to achieve tumor control while minimizing damage to the surrounding normal tissue. High dose conformity and steep dose fall‐off outside the target area are necessary to minimize irradiation of normal brain tissue.^1^, ^2^ Since the brain volume receiving 10 Gy (V_10Gy_) and 12 Gy (V_12Gy_) in stereotactic radiosurgery (SRS), and V_25Gy_ and V_30Gy_ in five‐fraction stereotactic radiotherapy (SRT) have been reported to be associated with radiation‐induced brain necrosis, it is important to reduce these parameters during radiotherapy planning.^3^, ^4^ However, it is important to deliver sufficiently high doses over a wide area of the gross tumor volume (GTV) to improve local control of tumors.^5^, ^6^ Some studies have reported that the inhomogeneous dose distribution of GammaKnife (GK) showed better local control than the homogeneous distribution of the LINAC‐based SRT.^7^, ^8^, ^9^

The optimization of IDL has an impact on dose distribution and normal tissue sparing, but, the IDL in cranial SRS or SRT varied substantially among institutions due to differences in modalities and treatment policies.^10^ Currently, advances such as multi‐leaf collimators and image‐guided radiation therapy (IGRT) have made it possible to achieve high dose compliance and treatment efficiency using volumetric modulated arc therapy (VMAT) technology.^11^, ^12^ However, the IDL in GK was generally around 50%, while LINAC‐based SRS usually used a higher IDL of 80%−90%.^13^ Studies on optimal IDL selection in intracranial SRS or SRT reported 50%−80% for dynamic conformal arcs (DCA) and 60%−70% for VMAT for brain dose reduction and planning target volume (PTV) dose coverage.^10^, ^14^, ^15^, ^16^

A new commercially available stereotactic treatment approach, named HyperArc (Varian Medical Systems, Palo Alto, CA), was recently released. In stereotactic irradiation for brain metastasis, HyperArc can achieve higher conformity and steeper dose‐fall off than conventional VMAT.^17^ Vergalasova et al. reported that HyperArc provided similar or better low‐dose spread than GK, while maintaining excellent conformity and minimizing inter‐planer variability.^18^ Hence, the treatment planners at our institute set out to investigate the optimal IDL in HyperArc theat has the potential to create a steeper dose distribution than manual VMAT. There is no report on the optimal IDL in HyperArc treatment yet. Since the clinical use of HyperArc in radiotherapy of brain metastases is on the rise and will become more widespread, clarifying the optimal IDL for SRT using HyperArc will help many planners in their clinical work.

The objective of the study was to compare the dosimetric parameters for target and normal brain tissue among the six plans with different IDLs and to estimate the optimal IDL for brain metastasis using HyperArc. Due to the increased complexity of dosimetric analysis in cases with multiple metastases, this study focused on single‐lesion cases. Following local practice, SRT with five fractions was employed in this study.

## 2 METHODS

### 2.1 Patients

The study protocol was approved by the ethics committee of the Osaka International Cancer Institute (OICI). All patients provided written informed consent. This study included 30 patients with single brain metastasis who received SRT treatment with five fractions between December 2019 and September 2021 at OICI. For each patient, computed tomography (CT) images for treatment planning were acquired using the CT scanner Revolution HD (GE Medical Systems, Milwaukee, WI) with an Encompass immobilization system (QFix, Avondale, PA). The scanning parameters were a matrix size of 512 × 512, a slice thickness of 1.0 mm, and a field view of 350 mm. The GTV was delineated on CT images, with a gadolinium‐enhanced T1‐weighted magnetic resonance imaging set. PTV was generated by adding an isotropic margin of 1.0 mm to GTV. The GTV ranged in volume from 0.01 to 28.53 cm^3^ with a median volume of 0.78 cm^3^. The 30 patients were divided into four groups based on GTV as follows,

1. Small group (*n* = 7): GTV ≤ 0.1 cm^3^
2. Medium group (*n* = 7): 0.1 cm^3^ < GTV ≤ 0.5 cm^3^
3. Large group (*n* = 7): 0.5 cm^3^ < GTV ≤ 5.0 cm^3^
4. Very large group (*n* = 9): 5.0 cm^3^ < GTV

### 2.2 HyperArc planning

We used Eclipse treatment planning system version 15.6 (Varian) for HyperArc planning. All treatment plans were created on a TrueBeam Edge (Varian) equipped with high‐definition multi‐leaf collimators, with 2.5 mm width of the first 32 leaves from the central point and 5 mm width for the remaining. The prescribed dose was 35 Gy in five fractions and all the plans were normalized such that 95% of the PTV received the prescribed dose. With HyperArc, the software automatically sets the optimal single isocenter, collimator angle, and non‐coplanar settings, taking into account the size and positioning of the targets, to provide a conformal plan with low dose to the normal brain tissue. The arc geometry (four arc fields: a full coplanar arc field with a 0° couch and three half‐non‐coplanar arc fields with a 45°, 315°, and 90°, or 270° couch) was automatically determined.^19^ All treatment plans were generated using 6‐MV photon beams with flattening filter‐free at a maximum dose rate of 1400 monitor units per minute. Dose optimizations and calculations were performed using a photon optimizer version 15.0 and an analytical anisotropic algorithm with a grid size of 1.25 mm, respectively.

**FIGURE 1:**
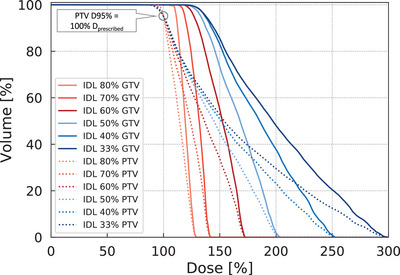
The DVH of GTV and PTV with varying IDL for one patient. The prescribed dose was 35 Gy in five fractions and all the plans were normalized such that 95% of PTV received the prescribed dose. The patient's GTV size was 0.86 cm^3^. DVH, dose volume histogram; GTV, gross tumor volume; IDL, isodode line; PTV, planning target volume. D_prescribed_, prescribed dose.

Six different retrospective HyperArc plans were generated for each of the 30 cases using approximately 80%, 70%, 60%, 50%, 40%, and 33% IDLs. Figure 1 shows examples of dose volume histograms (DVH) for six plans with varying IDL. Dose tuning structures were created for use in the optimization process. These structures were used to increase the central dose by shrinking the low dose spread outside the target. The tuning structures were created as four concentric volume rings or enclosures of various radii extending from the PTV as follows:

1. Ring1: inner = 0 mm from PTV; outer = 3 mm from PTV.
2. Ring2: inner = 3 mm from PTV; outer = 6 mm from PTV.
3. Ring3: inner = 6 mm from PTV; outer = 9 mm from PTV.
4. Ring4: inner = 9 mm from PTV; outer = 12 mm from PTV.

By adjusting the optimization parameters, six independently optimized plans were created with the IDL changed to satisfy D95% = 100% doses to maintain similar conformity. We did not use the Treatment Isodose Percent functionality provided in Eclipse as a way to change the IDL. Manual specification of the optimization objective during the optimization process was performed only for GTV and tuning structures. The optimization was performed by specifying the maximum dose for tuning structures and the lower objective for GTV, so that the DVH curve of GTV is linear. To simplify the study design, we did not set extra upper objectives to force up the dose inside the GTV, nor did we intentionally set lower objectives for the normal brain to suppress the spread in the mid‐dose range. SRS normal tissue objective (SRS‐NTO) was used to suppress the spread of the dose distributions. It controls the dose fall‐off outside the targets and dose bridging between targets. SRS‐NTO is automatically set for HyperArc plans which recognizes the spatial arrangements of targets for which dose bridging is likely to occur and prevents it from occurring at least at dose levels higher than 17% of the prescriptions.

### 2.3 Evaluation methods

Several dose parameters were used to evaluate the optimal IDL in the HyperArc plans. The gradient index (GI) was used to quantify the dose fall‐off as follows:

$$\mathrm{GI}\;=\;\frac{V_{0.5\mathrm{PD}}}{V_{\mathrm{PD}}}$$

where, *V*_0.5PD_ is the volume receiving 50% of the prescription dose and *V*_PD_ is the volume receiving the prescription dose.^20^ The smaller the GI of the plan, the sharper the dose fall‐off, indicating less spread in the distribution. *V*_25Gy_ and *V*_30Gy_ of brain minus GTV (brain–GTV) values were also evaluated as doses related to brain necrosis.^4^ The doses covering 99% (*D*_99%_) and 80% (*D*_80%_) of GTV were evaluated. The conformity index (CI) defined by Paddick^21^ was also calculated.

$$\mathrm{CI}\;=\;\frac{PTV_{PD}^{2}}{PTV\times V_{PD}}$$

where, PTV_PD_ was PTV covered by the prescription dose.

The Wilcoxon signed‐rank test was used to calculate the significance of the dosimetric parameters among the different IDL plans. Statistical significance was defined at *p* < 0.05. Statistical analysis was performed using open‐source statistical computing packages NumPy, Pandas, and SciPy for Python 3.8, and Jupyter Notebook, an open‐source interactive computing platform.

## 3 RESULTS

Table 1 lists the characteristics of the patients and their brain metastasis. The median age of the patients was 66 years (range: 30−85 years). The patient's primary cancers were located in the lung, esophagus, stomach, kidney, colon, breast, and skin. The tumors were situated in the frontal lobe, temporal lobe, parietal lobe, occipital lobe, and cerebellum.

**TABLE 1:** Characteristics of patients treated with five‐fraction SRT.

Characteristic	Value
Patients (*n* = 30)	
Age	
Median	66
Range	30−85
Sex	
Female	9
Male	21
Primary cancer	
Lung	19
Esophagus	3
Stomach	2
Kidney	2
Colon	2
Others	2
Tumor location	
Frontal lobe	10
Temporal lobe	5
Parietal lobe	3
Occipital lobe	7
Cerebellum	5

Abbreviation: SRT, stereotactic radiotherapy.

Six plans were generated for the medium, large, and very large volume groups but few generated plans were able to achieve an IDL ≤ 60% for the small volume group. Out of seven cases in the small volume group, only six cases could have plans with IDL of 60% and 50%, three cases with IDL of 40%, and two cases with IDL of 33%.

**FIGURE 2:**
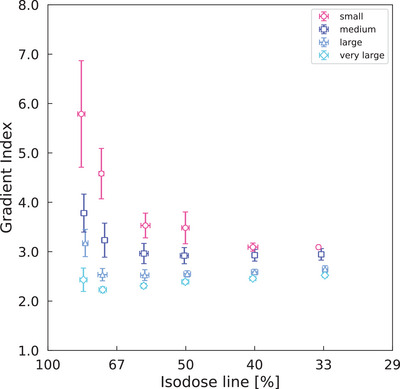
GI versus isodose line for different tumor sizes. On both the vertical and horizontal axes, data points indicate mean values and error bars indicate SD. GI, gradient index; SD, standard deviation.

Figure 2 demonstrates that GI decreased continuously with decreasing IDL in the small volume group. However, in the medium, large, and very large volume groups, GI initially decreased with decreasing IDL, reached a minimum, and then increased gradually. In contrast, IDL with minimal GI was observed between 1.0 and 3.0 for all groups except the small volume group. The IDL was lower for smaller tumors and higher for larger tumors.

**FIGURE 3:**
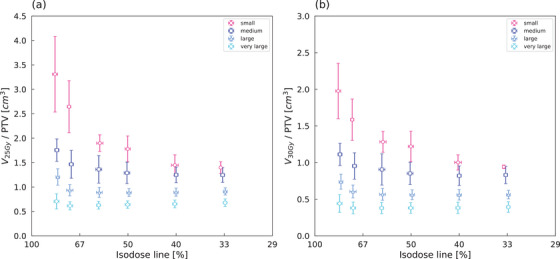
The ratio of brain–GTV volumes receiving (a) 25 Gy and (b) 30 Gy to PTV (V_25Gy_/PTV and V_30Gy_/PTV) versus IDL for different tumor sizes. On both the vertical and horizontal axes, data points indicate mean values and error bars indicate SD. GTV, gross tumor volume; IDL, isodose line; PTV, planning target volume; SD, standard deviation.

**FIGURE 4:**
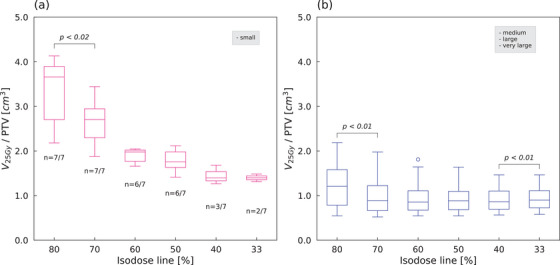
The boxplots of the ratio of brain–GTV volumes receiving 25 Gy to PTV (V_25Gy_/PTV) for (a) small volume group and (b) medium, large, and very large volume groups. GTV, gross tumor volume; PTV, planning target volume.

The ratio of brain–GTV volumes receiving 25 Gy (V_25Gy_/PTV) and 30 Gy (V_30Gy_/PTV) to PTV also showed the same trend as GI (Figure 3). Figure 4 shows boxplots of V_25Gy_/PTV of brain–GTV in the small volume group and medium to very large volume groups. A significant difference test performed on the small volume group showed a significant difference between 80% and 70% plans. In the combined medium to very large volume groups, the V_25Gy_/PTV was lower for 70% plans than 80% plans (*p* < 0.01), and higher for 33% plans than 40% plans (*p* < 0.01). No significant differences were observed for 70% and 40% of the plans. Patients with a GTV less than 19.0 cm^3^ met Andrsuka's threshold for an absolute brain dose of V_25Gy_ < 16.0 cm^3^.

**FIGURE 5:**
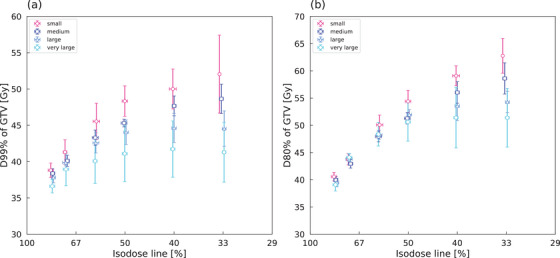
Dose that covers 99% (D_99%_) and 80% (D_80%_) of GTV versus isodose line for different tumor sizes. On both the vertical and horizontal axes, data points indicate mean values and error bars indicate SD. GTV, gross tumor volume; SD, standard deviations.

As shown in Figure 5a, the mean values of D_99%_ for GTV increased (37.8, 40.0, 42.6, 44.3, 45.1, and 45.1 Gy) as IDL decreased from 80% to 33%. The rate of increase in *D*_99%_ was higher with smaller target sizes. The mean *D*_99%_ increased from 38.8 to 52.1 Gy in the small volume group with a decrease in IDL from 80% to 33%, while the increase was from 36.6 to 41.3 Gy in the very large volume groups. The standard deviation (SD) in D_99%_ increased with a lower IDL, regardless of target size. The *D*_80%_ of GTV showed a similar trend to *D*_99%_ (Figure 5b). The means (SD) of D_80%_ were 39.7 Gy (1.0 Gy), 43.7 Gy (0.9 Gy), 48.6 Gy (1.7 Gy), 51.9 Gy (2.6 Gy), 54.1 Gy (4.4 Gy), and 55.1 Gy (5.2 Gy) for IDL 80% to 33%, respectively.

CI values did not show IDL dependence. All plans were normalized such that 95% of PTV received the prescribed dose, so the ideal CI was 0.95. The mean (SD) of CIs in the medium, large, and very large volume groups was 0.93 (0.03), 0.92 (0.03), 0.92 (0.05), 0.92 (0.04), 0.93 (0.03), and 0.93 (0.03) for IDL ranging from 80% to 33%. In the small volume group, CI tends to be smaller, with a mean (SD) of 0.75 (0.08), 0.77 (0.06), 0.81 (0.02), 0.81 (0.07), 0.87 (0.08), and 0.91 (0.04) for IDL levels from 80% to 33%, respectively.

## 4 DISCUSSION

The present study investigated the optimal IDL of the HyperArc planning for metastatic brain tumors by comparing six plans with different IDLs. The optimal IDL was concluded from two viewpoints: the lower V_25Gy_/PTV of Brain‐GTV and the D_99%_ of GTV exceeding 43 Gy. For GTVs larger than 0.1 cm^3^, IDLs that met the former requirement ranged from 40% to 70%, while those meeting the latter ranged from 33% to 50%. Thus, considering both reduction of normal brain dose and local control of tumor, IDL 40%−50% is the optimal IDL for GTV larger than 0.1 cm^3^. This optimal IDL was lower than that of DCA and VMAT. Since HyperArc is one of the most effective radiotherapeutic modalities for brain metastases, our study findings would further assist planners in determining dose distribution inhomogeneity in the future.

The relationship between IDL and GI or brain dose was similar to that observed in previous studies.^14^, ^15^ The optimal IDL depended on tumor size, in particular, for GTV volumes above and below 0.1 cm^3^. Andruska et al. suggested that V_25Gy_ and *V*_30Gy_ of uninvolved brain are important for evaluating five‐fraction SRT to minimize the risk of radiation necrosis.^4^ Since our institution also considers it important to lower the *V*_25Gy_ and *V*_30Gy_ of Brain‐GTV as much as possible and employs these values as factors to conclude plan quality, we also used them in determining the optimal IDL. According to our results, the optimal IDL for significantly lower *V*_25Gy_/PTV was less than 60% for GTV volumes less than 0.1 cm^3^ and 40%−70% for GTV volumes larger than 0.1 cm^3^. Whether Andruska's threshold is met depends more on the GTV size than on how low the IDL is. In fact, only three patients in our study with GTV sizes of approximately 19.0 cm^3^ or more, exceeded the threshold for brain dose, *V*_25Gy_ < 16.0 cm^3^. In such cases, it is important to create a dose distribution that reduces the radiation necrosis risk by selecting the optimal IDL. Even if the GTV were small enough to not exceed this threshold, it is still worthwhile to select the optimal IDL to reduce the dose to the brain‐GTV as much as possible.

Low IDL plans that can increase the target dose with an inhomogeneous dose distribution may be effective in improving local tumor control.^7^, ^8^, ^9^, ^22^ Dupic et al. identified that the GTV D_98%_ was a strong reproducible significant predictive factor of local control for brain SRT.^5^ The association between the biological effective dose (BED) using a linear‐quadratic model and local tumor control has been reported in several studies. Matsuyama et al. reported that the 1‐year local control rate was significantly better if the lesions received the BED that assumed α/β of 10 (BED10) 80 Gy or higher.^23^ Kanayama et al. suggested that increased *D*_80%_ of GTV with heterogeneous dose distribution is crucial for better local control.^6^ Therefore, it is important to deliver a sufficient dose to the GTV as widely as possible for local tumor control. This study showed that the IDL should be less than 50% to cover BED10 80 Gy(43 Gy/five fractions)in 99% of the GTV volume. However, it should be that GTV D_99%_ was also dependent on tumor size, and hence, the larger the GTV volume, the more difficult it was to increase the GTV minimum dose. In addition, good local control might require higher *D*_80%_ of GTV, and an inhomogeneous dose distribution below 50% IDL would be desirable for *D*_80%_ to exceed 50 Gy in five fractions. Tumor shape is also important in the selection of IDL. An inhomogeneous dose distribution with a low IDL would result in a near spherical distribution in three dimensions, increasing the likelihood of high dose hits outside the targets with complex shapes. Therefore, one must be cautious while choosing low IDLs for complex shaped tumors. Furthermore, IGRT should be utilized during treatment and daily tumor volume changes should be carefully monitored.

In cases with a GTV volume of 0.1 cm^3^ or less, lower IDL resulted in a lower dose to the brain and a higher dose to the GTV. However, some cases in our study could not achieve an IDL of 60% or less, and hence the optimal IDL could not be determined. This is the limitation of treatment planning systems while calculating doses for very small fields, in situations where the field aperture is smaller than the minimum beam model input data of the treatment planning system. While planning for small targets, the radiation dose to the brain was not high enough to cause brain necrosis, except in a few cases with a threshold concern. Furthermore, a homogenous distribution is sufficient for tumor control in small tumors.^24^ It is appropriate to consider the risk of misalignments in small target to achieve an unreasonably low IDL.

In a previous study that showed the optimal IDL in VMAT, five different plans with varying IDLs　(50%, 60%, 70%, 80%, and 90%) were created.^16^ Hence, we hypothesized that HyperArc could be used to create more inhomogeneous dose distributions, and we decided to generate plans with lower IDLs than those in previous studies. In fact, for GTVs larger than 0.1 cm^3^, a plan with IDL 33% could be generated, and HyperArc showed no significant increase in brain dose even at an IDL of 40%, where brain dose turned upward in VMAT. If the IDL is smaller than 60% in VMAT, the sharpness of the dose fall‐off reaches its limit, and a higher global maximum dose may result in irradiating more of the surrounding normal brain tissues. This limitation was overcome by using HyperArc to create an even steeper distribution owing to the powerful dose reduction capability of SRS‐NTO. As a result, it was possible to achieve GK‐like inhomogeneity while keeping the brain dose low. However, it should be noted that the resolution of the specific patient quality assurance (QA) tool may not be fine enough for such an irregularly heterogeneous distribution. Usage of appropriate patient QA methods must be considered.

There were a few limitations to this study. First, the number of plans to determine the optimal IDL was small. There were only seven or nine plans per group when grouped according to GTV size. A larger sample size would have provided more reliable results. The lack of data for the small group was particularly noticeable. Second, this study included only cases with single metastasis with nearly spherical tumor shape and located far away from critical organs at risk and skull. More complex considerations would be necessary if multiple metastatic lesions were irradiated at once, or if the brainstem or nearby areas were irradiated, or if tumors with complex shapes were targeted. Future research is needed, especially with the advent of HyperArc, to irradiate multiple metastatic lesions at once. Third, optimization objectives were set only for GTV and tuning structures during treatment planning. In this study, the treatment plans were created for the DVH curve of the GTV to be linear. Although it would be possible to create a plan with an even lower brain dose by directly applying the appropriate optimization objective to brain‐GTV, the essence of the present study was to investigate the homogeneity of dose distribution and the dose fall‐off relationship.

## 5 CONCLUSION

This study investigated the optimal IDL in HyperArc plans for brain metastasis. The optimal IDL depended on tumor size, with an optimal IDL of 40%−50% for GTV volumes over 0.1 cm^3^. The study's finding helps treatment planners determine the IDL for cranial SRT using HyperArc. It is possible to create a plan with an IDL as low as that in GK using HyperArc, and in fact, this study reported that the optimal IDL was lower than that in DCA and VMAT.

## AUTHOR CONTRIBUTIONS

All authors (1) made substantial contributions to the study concept or the data analysis or interpretation; (2) drafted the manuscript or revised it critically for important intellectual content; (3) approved the final version of the manuscript to be published; and (4) agreed to be accountable for all aspects of the work.

## CONFLICT OF INTEREST STATEMENT

The authors declare that there are no conflicts of interest.

## Data Availability

Research data are stored in an institutional repository and will be shared upon reasonable request to the corresponding author.
